# Weight-control behaviour and weight-concerns in young elite athletes – a systematic review

**DOI:** 10.1186/2050-2974-1-18

**Published:** 2013-05-30

**Authors:** Anne Werner, Ansgar Thiel, Sven Schneider, Jochen Mayer, Katrin E Giel, Stephan Zipfel

**Affiliations:** 1 Department of Psychosomatic Medicine and Psychotherapy, University Hospital Tübingen, Osianderstr, 5, 72076, Tübingen, Germany; 2 Institute of Sports Science, Tübingen University, Tübingen, Germany; 3 Mannheim Institute of Public Health, Social and Preventive Medicine, Medical Faculty Mannheim, Heidelberg University, Mannheim, Germany

**Keywords:** Young elite athlete, Weight-control behaviour, Weight concern, Leanness sports

## Abstract

Weight-control behaviour is commonly observed in a wide range of elite sports, especially leanness sports, where control over body weight is crucial for high peak performance. Nonetheless, there is only a fine line between purely functional behaviour and clinically relevant eating disorders. Especially the rapid form of weight manipulation seems to foster later eating disorders. So far, most studies have focussed on adult athletes and concentrated on manifest eating disorders. In contrast, our review concentrates on young athletes and weight-control behaviour as a risk factor for eating disorders.

An electronic search according to PRISMA (Preferred Reporting Items for Systematic Reviews and Meta-Analyses) Statement was performed using Pubmed, PsychInfo and Spolit. The following search terms were used: *weight*-*control*, *weight*-*control behaviour*, *weight gain*, *weight loss*, *pathogenic weight*-*control behaviour* and *weight*-*concerns*, each of them combined with *elite athlete*, *young elite athlete*, *adolescent elite athlete* and *elite sports*.

Overall, data are inconsistent. In general, athletes do not seem to be at a higher risk for pathogenic weight concerns and weight-control behaviour. It does seem to be more prevalent in leanness sports, though. There is evidence for pathogenic weight-control behaviour in both genders; male athletes mostly trying to gain weight whereas females emphasise weight reduction. There is not enough data to make predictions about connections with age of onset.

Young elite athletes do show weight-control behaviour with varying degrees of frequency and severity. In particular, leanness sports seem to be a risk factor for weight manipulation. Further research is needed for more details and possible connections.

## Review

### Introduction

Weight-control behaviour is accepted in a wide range of elite sports [1,2]. Many sports require “low body weight and/or low fat/muscle ratio (leanness)” to achieve good results ([1] page 5, [3]). Quite often, this causes problems in terms of pathogenic weight-control behaviour or eating disorders. Research into adult elite athletes so far has suggested that pathogenic weight-control behaviour, disordered eating or clinically relevant eating disorders are common in elite sports [2,4-12]. However, there is much less evidence on these aspects in young elite sports.

For many athletes across various sport disciplines there is a direct causal connection between success and low body weight [13], with weight regulation playing a decisive role. Thus, Dosil classified so-called “high risk sports in connection with pathogenic weight-control behaviour” [2]. These are: 1. aesthetic sports, e.g. rhythmic and artistic gymnastics, ice-skating or dancing; 2. weight division sports, e.g. judo or rowing; 3. gym sports, e.g. aerobics; 4. endurance sports and 5. low-weight performance sports, e.g. distance running. In general athletes in these high-risk sports emphasize their slim appearance [2,3,13].

Weight-control behaviour can be pathogenic or non-pathogenic. Pathogenic weight-control behaviour is used to “manage emotions, weight and body size” [14] hence introduces a second dimension, namely feelings. This clearly distinguishes it from the non-pathogenic weight-control, which is purely functional and targeted in line with sport specific demands. Pathogenic refers to all unhealthy ways of controlling weight and can be carried out in two ways: gradual and rapid [10,15]. Gradual weight-control behaviour is a method of reducing or gaining weight in a more careful, continuous way, for example through restraint, selective eating or exercising over a longer period of time. Nonetheless, it can get out of control and might lead to sub-clinical eating disorders, with excessive exercising or long lasting dieting being risk factors for eating disorders [8,16,17]. On the other hand, rapid weight-control is characterized by a fast change of body weight through compensatory behaviour over a shorter time period (e.g. vomiting, obsessive sweating through sauna in plastic suits or using medication like laxatives). It might also – in connection with the pressure to be thin for a better athletic performance – increase the risk of developing an eating disorder [4,7,8,10,15,18-21]. In general, it is well recognized that dissatisfaction with either shape or weight of the body and a resulting dieting behaviour are risk factors for the development of eating disorders [22,23].

Elite athletes form a special population, as they participate in a highly competitive system, in which the main evaluation criterion is “victory or defeat” in sport competitions and sporting events [24]. Therefore, the main orientation is towards peak performances and outstanding bodily achievements. In order to attain this, elite athletes from almost all sport disciplines must focus on their body in some way [8]. This clearly distinguishes them from non-athletes and, consequently, has to be taken into account when evaluating the effect of amount of training, weight loss methods, eating patterns or psychological “profile” [8,20]. Sport specific weight-control behaviour can be purely functional and necessary for performance improvement, particularly in leanness-sports [25], making it a non-pathogenic form of weight-control. This means that the very same behaviour (e.g. restriction in eating) might be pathological in one context and non-pathological in another. However, the line between functional, non-pathogenic and sub-clinical pathogenic behaviour might be thin. Additionally, young athletes might be under double pressure, as they are not only under the normal strain from society, but the sport-specific demands on top [18].

Over the last decades, several studies and reviews have shown a higher prevalence of eating disorders among athletes compared to non-athletes. In contrast, this review will focus on pathogenic weight-control behaviour (e.g. restricted/disordered eating, abuse of medication) and weight-concerns (e.g. body dissatisfaction) rather than manifest eating disorders in elite athletes. We defined “elite athletes” as those actively competing at minimum in the national teams of their respective home country and/or being part of the national squad team. Additionally, we decided to focus on young elite athletes aged 12 to 25, as there is a substantial gap of knowledge on eating and weight-management behaviour in young and adolescent elite athletes. It has been reported that athletes start to manipulate their weight as early as aged 9 to 14 [26,27]. Empirical evidence suggests that the onset of eating and weight problems in the general population often happens during adolescence leaving young athletes potentially at double risk [28]. As young elite athletes often still attend college and educational systems vary in different countries, we decided to set the upper age limit to 25.

Therefore, the aim of this review is to have a closer look at weight-control behaviour in young elite athletes, providing a comprehensive overview of the data published to answer the following questions:

1. Are young elite athletes more at risk for pathogenic weight-control behaviour than non-athletes? Is there some kind of functionality for (pathogenic) weight-control behaviour in young elite sports?

2. Are young elite athletes competing in leanness-sports more at risk for pathogenic weight-control behaviour than athletes competing in non-leanness sports?

3. Are young elite female athletes more at risk for pathogenic weight-control behaviour than young male athletes?

Answers could be used to develop special guidelines for dealing with the occurring weight-control behaviour in young elite athletes, similar to those already shown for adults [12].

### Methods

The systematic procedure of this literature review follows the PRISMA-Statement (Preferred Reporting Items for Systematic Reviews and Meta-Analyses) [29]. An electronic search was performed using Pubmed, PsychInfo and Spolit. Additionally, references from retrieved articles were examined for cross-references. The final database search was carried out on February 27, 2012. We applied no language or year restrictions. As one study eligible for our review only had a Norwegian full text, a bilingual research assistant translated the article.

Search strategy was performed by using search terms based on the Medical Subject Heading (MeSH) with 24 combinations performed in each database. The terms *weight*-*control*, *weight*-*control behaviour*, *weight gain*, *weight loss*, *pathogenic weight*-*control behaviour* and *weight*-*concerns* were each combined with *elite athlete*, *young elite athlete*, *adolescent elite athlete* and *elite sports*.

Inclusion criteria for the search procedure were that our search terms appeared either in title or abstract. Additionally, studies had to focus on (a) “elite” athletes (defined as athletes competing at minimum in the national teams of their respective home country and/or being part of a national selection squad), (b) athletes still actively competing at time of assessment, (c) young or adolescent athletes (defined as younger than 25 years on average including the standard deviation of the sample) and (d) the terms “weight concerns” or “weight-control behaviour” as primary outcome variable. Reviews and meta-analyses were also included. We excluded dissertations and essays.

Two blinded reviewers assessed the abstracts and full texts independently for inclusion and exclusion criteria. The inter-rater-reliability was κ =0.80 and 0.82, respectively, reflecting a good to very good agreement. In cases of disagreement, a common conclusion based on consent could be found.

### Results

**Figure 1:**
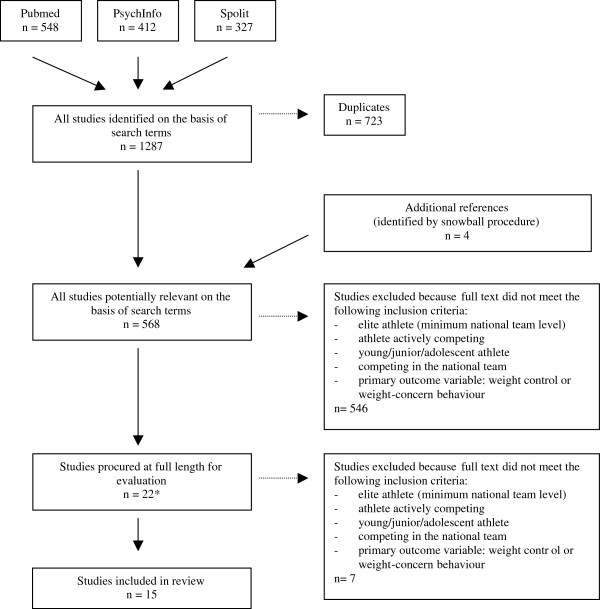
Flow-chart overview of literature search process.

After excluding duplicates, we found a total of 568 articles of which 15 were eligible for our review (see flow chart, Figure 1). Thirteen articles looked at “weight-control behaviour” or “pathogenic weight-control behaviour” as the primary outcome variable; only two articles looked at weight-control behaviour alone [4,19]. Another two studies focused on “weight-concerns” as the primary outcome variable [30,31], with one additionally looking at weight-control behaviour [31]. All studies eligible for inclusion were either in English or German. Table 1 gives an overview of all articles included in the review (see additional files). All results presented are significant unless stated otherwise.

**Table 1:** Overview of all studies included in the review

**Author**	**Age in years(mean, SD)**	**Gender**	**Sample size total**	**Sample size athletes**	**Sample size controls**	**Sports type**	**Competition level**	**Controls**	**Outcome**	**Instruments**	**Results**
Anderson et al., 2011 [18]	19.14 +/− 1.86	females only	414	414	0	gymnastics, swimming/ diving	NCAA Division-I	n/a	WC, WCB	Body Parts Satisfaction Scale, Dietary Intent Scale	No significant difference between athletes competing in leanness and non-leanness sports.
Arroyo et al., 2008 [30]	19.6 +/− 1.3	males only	56	28	28	soccer	Professional soccer team	age- and BMI-matched students; engagement in recreational sports <3hrs/week	WC	Somatomorphic matrix test	No significant difference between athletes and controls
Artioli et al., 2010 [4]	19.3 +/− 5.3 not specified according to gender	607 male, 215 female	822	822	0	judo	national and international	n/a	WCB	Rapid Weight Loss Questionnaire	Athletes were engaged in several forms of pathogenic weight control behaviour. No gender differences could be found. An earlier start of using weight-control methods leads to more aggressive variants.
Ferrand et al., 2005 [32]	athletes: 15.4 +/− 1.2 (swimmers) and 16.5 +/− 0.93 (rest) controls: 16.3 +/− 1.1	not specified; only the swimmers included males	132	82	50	synchron. swimming, non-leanness sports (basketball, handball, soccer, volleyball)	national	non-athlete college students; no further elaboration about their sports activities	WC, WCB	Canadian-French version of Body-Esteem Scale, French version of Eating Attitudes Test	Athletes showed more weight concerns but not more weight-control behaviour than controls. No significant difference between athletes competing in leanness and non-leanness sports.
Galli et al., 2009 [47]	23 +/− 0.68	males only	10	10	0	baseball, diving, football, golf, lacrosse, skiing, swimming	national and international	n/a	WC, WCB	Qualitative study using semi-structured interviews	Athletes did show some weight concerns.
Greenleaf et al., 2009 [19]	20.16 +/− 1.31	females only	204	204	0	basketball, cheerleading cross-country, field hockey, golf, gymnastics, rowing, skiing, soccer, softb., swimming, synchron. swimming, tennis, track&field, volleyball	NCAA Division-I	n/a	WCB	Adapted version of Questionnaire of Eating Disorder Diagnosis, Bulimia Test-Revised	No significant difference between athletes competing in leanness and non-leanness sports.
Johnson et al., 1999 [44]	19.9 20.1 (male), 19.6 (female); no SD given	883 male, 562 female	1445	1445	0	basketball, tennis, cross-country, football, gymnastics, nordic skiing, swimming, volleyball, wrestling	NCAA Division-I	n/a	WC, WCB	Self-created questionnaire including subscales from EDI-2, Rosenberg Self-Esteem Scale and Body Cathexis Scale	No significant difference between athletes competing in leanness and non-leanness sports. Female athletes have more pathogenic weight concerns and weight-control behaviour than male ones.
Marshall et al., 1996 [31]	20.8 +/− 3.8 (juniors 17.1 +/−0.9 and seniors 22.5 +/− 3.2)	not specified	111	111	0	field hockey	national and international	n/a	WC, WCB	EDI	Athletes did show weight concerns. No difference in pathogenic weight concerns and weight-control behaviour in connection with age.
Martinsen et al., 2010 [13]	15-16 (range) no mean given not specified according to gender	athletes: 389 male, 217 female controls: 197 male, 158 female	961	606	355	50 different sports types, classified into leanness and non-leanness sports	students at elite sport schools, no further elaboration of competition level	age-matched 1st year high school students; no further elaboration about their sports activities	WC, WCB	EDI-2	Controls used pathogenic weight-control behaviour significantly more often than athletes. Different reasons for weight control. No significant difference between athletes competing in leanness and non-leanness sports. Female athletes have more pathogenic weight concerns and weight-control behaviour than male
Parks and Read, 1997 [42]	14-18 (range) no mean given	males only	74	74	0	cross-country running, football	national	n/a	WC, WCB	Body Esteem Scale, Body Size Drawings, Eating Attitudes Test, Reason for Exercising Inventory	Athletes competing in leanness sports showed more pathogenic weight concerns and weight-control behaviour than athletes competing in non-leanness sports.
Pietrowsky and Straub, 2008 [41]	rowers: 22.00 +/− 2.00 (heavyweight) and 22.06 +/− 2.89 (lightweight) handball: 28.25 +/− 3.91	males only	164	132	32	rowing	national and international	non-athletes; engagement in recreational sports less than once a month; handball players from national team	WC, WCB	Silhouettes similar to the Body Image Assessment, Three-Factor Eating Questionnaire	Controls and athletes lightweight rowers had a more weight concerns if hungry, whereas heavyweight rowers and handball players showed more weight concerns in satiety. All athletes showed weight-control behaviour.
	non-athletes: 25.56 +/−4.47 (restraint eating group) and 28.00 +/− 4.29 (unrestraint eating group)										
Reinking and Alexander, 2005 [33]	athletes: 19.7 +/− 1.1	females only	146	84	62	swimming, cross-country, basketball, volleyball, soccer, softball, field hockey	NCAA Division-I	undergraduate students; no further elaboration about their sports activities other than “not athletes in collegiate sports”	WC, WCB	EDI-2	Controls showed more weight concerns and weight-control behaviour than athletes. Athletes competing in leanness sports showed more pathogenic weight concerns and weight-control behaviour.
	controls: 20.2 +/− 1.2										
Rosendahl et al., 2009 [34]	14-18 (range) no mean given	athletes: 366 male, 210 female controls: 122 male, 169 female	867	576	291	26 different sports: technical, endurance, aesthetic, weight class, ball game, power, antigrav. sports	national and international	students from non-Elite Sports Schools; no further elaboration about their sports activities	WC, WCB	Eating Attitude Test, Silhouettes	Controls more often showed a history of weight-control behaviour than athletes, only significant in females. Athletes competing in leanness sports scored higher for weight control than athletes competing in non-leanness sports. Gender differences in intention.
	not specified according to gender										
Rouveix et al., 2007 [35]	athletes: 16.5 +/− 0.5 (male) and 17.2 +/− 1.1 (female) controls: 21.8 +/− 1.8 (male) and 20.2 +/− 3.0 (female)	athletes: 12 male, 12 female	55	24	31	judo	national	random sample with participants not training more than 3hrs/week	WC, WCB	Self-administered questionnaire, French version of Eating Attitudes Test, Body Esteem Scale	No significant difference between athletes and controls concerning weight concerns. Significant difference in weight-control behaviour. There was a gender difference in used methods and ideal body.

		controls: 17 male, 14 female									
Thiel et al., 1993 [45]	21.1 +/− 2.4	males only	84	84	0	rowing, wrestling	national	n/a	WC, WCB	Self-created questionnaire, EDI-2	Athletes did not show pathogenic weight-control behaviour.

*WC* = weight concerns, *WCB* = weight-control behaviour.

### Weight-control behaviour in young athletes and non-athletes

According to cross-sectional cohort studies using questionnaires (e.g. Rapid Weight Loss Questionnaire, Eating Disorder Inventory) or standardized clinical interviews, young elite athletes are not more at risk for developing or showing pathogenic weight concerns or weight-control behaviour than non-athletes [13,30,32-35].

Two studies found no difference between controls and athletes [30,35]. Arroyo et al. [30] examined 56 elite soccer players and 28 age- and BMI-matched non-elite athlete controls with regard to body perception and satisfaction. In both groups around one fifth desired a higher weight and a more muscular appearance. The only difference, though not significant, in perceived body image between athletes and controls was that the soccer players perceived their body sizes smaller than the control group. Rouveix at al [35] compared judo athletes with non-athletic controls. Overall, there were some differences between athletes and controls with regard to weight concerns. Female athletes were less satisfied with their actual weight and wanted to have a lower body weight than their respective controls. Additionally, female athletes had more and higher fluctuations in their weight. However, all differences in satisfaction and perception were not significant. Nonetheless, the authors could show a significant difference in weight-control behaviour. They found, that one-quarter of the examined judo athletes but none of the controls showed a higher risk for pathogenic weight-control measured with the Eating Attitudes Test [36].

Three other studies found a higher prevalence of weight concerns and/or weight-control behaviour in controls than in athletes [13,33,34]. Martinsen et al. [13] examined 606 elite athletes from 50 different sports types (categorized into leanness and non-leanness sports) and compared them with an age-matched control group of 355 high school students. They found pathogenic weight-control behaviour (defined as use of diuretics, laxatives, vomiting and diet pills) more frequently in controls than in athletes. Additionally, there was a different reason for weight-control in male subjects: whereas male controls wanted to improve their appearance, male athletes tried to improve their performance. Rosendahl et al. [34] looked at weight concerns in 567 athletes of elite sports schools compared to 291 non-athlete high school pupils using the Eating Attitude Test [37] and silhouettes for body perception [38]. When looking at disordered eating and dietary experience as prior weight-control behaviour, athletes scored significantly lower than non-athletes with only the difference between female participants being significant. Reinking and Alexander (30) examined 84 female NCAA Division I athletes (16 competing in leanness and 68 in non-leanness sports) and compared them to 62 non-athletes using the Eating Disorder Inventory 2 [39]. All females had a lower mean desired body weight with athletes scoring significantly lower on the Body Dissatisfaction subscale than non-athletes. When assessing the risk for disordered eating, again non-athletes scored significantly higher than athletes.

One study came partly to the conclusion that athletes are more at risk for weight concerns but not weight-control behaviour [32]: Ferrand et al. compared 42 synchronized swimmers and 40 other athletes competing in “non-weight dependant” sports types with a control group of 50 students using the Canadian-French version of Body-Esteem Scale [40] and the French version of the Eating Attitudes Test [36]. Synchronized swimming athletes had more weight concerns than the female control subjects but showed no difference on weight-control behaviour subscales.

Finally, a study by Pietrowsky et al. [41] with male light- and heavy-weight rowers from the German national team examined the body dissatisfaction in connection with leanness and muscularity under hunger and satiety conditions and compared them to handball players and non-athletes using male body silhouettes. Their findings are heterogeneous: As a result, the examined athletes struggle with weight problems, control of eating behaviour and body dissatisfaction, especially those competing in the lightweight group. This result varied between the groups as lightweight rowers and non-athletes had greater body dissatisfaction when hungry, whereas it was the other way round with heavyweight rowers and handball players.

When looking at the data found, several points have to be considered. All studies included used self-reported measures, which are prone to distortion. Two studies [30,34] complemented questionnaires with silhouettes to determine athletes’ weight concerns in order to strengthen their statement. However, the study of Rosendahl et al. raises major concerns [34]. The authors did not clarify at which level their “elite” athletes compete, in particular there were 101 athletes described as currently not belonging to a specific team. Additionally, there was no definition of the sports activities in the comparison group, so that any comparative conclusions must be drawn with care. Most worryingly, the two groups (athletes versus non-athletes) were already significantly different in their basic demographic characteristics. A lack of clear definitions appeared in two more studies, with neither elaborating what the control groups’ sports activities were [13,32].

In contrast, the study by Reinking et al. did not clearly distinguish between their athletes and controls [33]: 43.6% of the control group participated in sports activities 4 times per week and more, which makes the distinctive feature of the athlete group questionable. Furthermore, only athletes from one year at one university have been examined, and these were a “convenience sample”, with athletes voluntarily joining after an information session, which may have prevented students with severe weight-control behaviours or disturbed body images from participating. It remains unclear, therefore, how generalizable the results are.

Although the non-athletes in Pietrowsky et al.’s study were well controlled with regard to their sport activity (less than once a month) their results must also be interpreted with care [41]. Only the light- and heavyweight rowers were within our category of “young athlete” whereas the other participants all belonged to older age groups. This makes comparisons beyond the different rower groups unreliable. A very specific problem resulted from the sample composition of Ferrand et al.’s study [32]: One of their subgroups, swimmers, consisted of female and male athletes whereas the two other groups were female samples only. Any result found could thus be due to gender characteristics rather than sports type specific or control group related.

A further weakness of many of the studies was sample size. For example, both Arroyo et al. and Martinsen et al. had a substantial difference in group sizes between athletes and controls, which might distort the results [13,30]. Two other studies [35,41] only examined very smalls groups so that it is hard to determine how valid results are even if relatively specific instruments were used [35].

Summarizing these data, larger studies in particular seem to suggest that young elite athletes are not more at risk for pathogenic weight concerns and weight-control behaviour than non-athletic controls. Participating in elite sports might even be a protective factor. However, due to the overall limited number of studies, with simultaneously dozens of different sport types to be examined there is not enough data in total. Additionally, several methodological problems across the different studies make a clear final conclusion impossible.

### Weight-control behaviour in connection with type of sport

Three studies suggest that restrict weight-control is particularly prevalent in leanness-sports defined as sports emphasising leanness or low body weight (such as aesthetic or endurance sports) [33,34,42]. Reinking and Alexander [33] could show that within their NCAA Division I athletes, those competing in leanness sports scored significantly higher on the sub score for Body Dissatisfaction than those competing in non-leanness sports. Equally, athletes from leanness sports had a lower mean desired body weight than athletes from non-leanness sports. Parks et al. [42] compared 30 male cross-country runners with 44 male football players. They could show a significant difference between these two groups, with runners expressing more weight concerns and aiming for less weight than footballers who wanted to if anything gain weight. Rosendahl at al [34] could show in addition to the comparisons with controls mentioned above that athletes competing in leanness sports scored higher for weight-control behaviour than those competing in non-leanness sports.

In contrast, four other studies did not find any differences between leanness- and non-leanness sports weight-control behaviour [13,18,19,32]. Greenleaf et al. [19] were looking at 204 NCAA Division I athletes of various sports types from 3 different universities using the Questionnaire for Eating Disorder Diagnosis [43]. As a result, over half of the athletes (54.4%) were dissatisfied with their weight. The majority of them wanted to lose weight, on average around 5.9 kg. Additionally, there was a weight fluctuation of more than 10% within a year in 1/5^th^ (in season) to 1/3^rd^ (out of season) of the athletes. 25.5% admitted to exercise at least 2 hours a day specifically to reduce their weight, 15.7% were fasting or on a strict diet for the very same reason. However, only a small minority indicated purging behaviour: 2.9% reported vomiting 2–3 times/month, another 1.5% taking diuretic medication 2–3 times/month and 1% using laxatives 1–2 times/week. For all weight concerns and weight-control behaviours, no difference could be found between the various types of sports. Ferrand at al compared weight-concerns in 42 synchronized swimmers and 40 other “non-weight dependant sports types” (e.g. basketball, volleyball, soccer), finding no difference [32]. Martinsen et al. [13] in their study with 606 elite athletes from 50 different sports types showed similar frequencies of pathogenic weight-control methods such as vomiting, laxatives, diuretics and diet pills. 11.1% of the female and 2.1% of the male athletes used pathogenic weight-control methods. When looking at weight concerns and the feeling of being “too fat”, 24% of the female athletes and 7.5% of the male ones were dieting now and/or have been on a diet at least three times before due to this reason. Johnson at al [44] could show some differences between different types of sports. However, this was only true in two instances and with no particular connection to the categories of leanness and non-leanness sports: In female athletes gymnasts scored higher on the Drive for Thinness Scale than swimmers and basketball players. Male football players showed greater body dissatisfaction than gymnasts and cross-country athletes.

In their study about psychosocial correlates of bulimic symptoms, Anderson et al. used structural equation modelling to test pathways and constructs in 280 NCAA Division I female gymnasts and 134 NCAA Division I female swimmers/divers from 26 universities [18]. They could show that the level of body dissatisfaction as well as restrictive eating was related to the amount of experienced pressure from teammates and coaches. There was no relation between the two components themselves. No difference with regard to type of sport could be shown.

Looking at weight-dependent sports only, two studies described methods of rapid weight-control amongst judo athletes [4,35]. A large survey by Artioli et al. [4] exploring prevalence and patterns of rapid weight loss among male and female judo competitors shows that most elite judo athletes (86%) have reduced weight rapidly before competition. Most of them regained this lost weight at least partially within one week after competition. The average magnitude of weight reduction was around 2-5% of their body-weight, with some athletes loosing up to 10% ten times or more in their careers. The fluctuation of weight was confirmed by a study from Rouveix et al. showing that rapid weight reduction is an inherent part of the lives of judo athletes [35]. In their study, they could show that during a season 70% of the athletes lost more than 2.8 kg, primarily by increasing exercises or limiting the intake of food and fluids. Nearly two thirds limited their choice of food on a constant basis.

Other studies examined only selected sports types [31,45] describing the prevalence of weight concerns and weight-control behaviour in a particular field. Thiel et al. [45] deliberately chose male athletes of lower weight categories to match them to female athletes from ballet and dancing previously identified as high-risk groups for subclinical eating disorders. The majority of athletes did not score pathologically for weight concerns and weigh-control behaviour. In particular, there was no difference between the male rowers and wrestlers. Only if they formed a subgroup of athletes with the diagnosis of a subclinical eating disorder based on the EDI-2 [39] Drive for Thinness subscale scores, did they find a difference on Body Dissatisfaction scale in comparison to the rest of the subjects. Marshall at al examined 120 female Canadian elite field hockey players [31]. In general, only a very low number of athletes (3.65%) scored pathological on the Drive for Thinness scale of the Eating Disorder Inventory, thus indicating a preoccupation with weight. However, they did find a fivefold higher frequency of athletes scoring at risk for the Body Dissatisfaction (BD) scale, which made the authors conclude a higher prevalence of weight concerns. Interestingly, athletes with high BD scores could be shown to be indeed heavier and with more body fat than their colleagues so there might be some truth in the athletes’ perceptions.

Rouveix et al. included a comparably small sample size of only 24 athletes [35]. Reinking et al. on the other hand had a substantial difference in group sizes with only 16 athletes competing in leanness sports as opposed to 68 athletes competing in non-leanness sports [33]. Thiel et al. also showed a significant difference in the group sizes of athletes with only 25 wrestlers but 59 rowers, which further reduce the generalizability of results found [45]. This study raises additional concern because they found differences only after creating a subgroup scoring pathologically for subclinical eating disorders, which makes their conclusions questionable [45].

In contrast, two studies used large, balanced samples [4,44]. A specific problem of the study by Johnson et al. was that large parts of their results were based on a self-created, unvalidated questionnaire that had its main focus on eating disorders [44]. Anderson et al. tried to make their results more generalizable by using a variety of universities to get their sample from which clearly reduces local biases [18]. Again, a lack of clear definitions made interpretation difficult: Two studies did not state clearly at which level their athletes competed [32,34]. In the study of Ferrand et al. [32] only the swimmers were classified as “elite” whereas the other athletes only were referred to as “national” ones without any further specification of the competing level. Additionally, the sample of swimmers consisted of female and male athletes whereas the two other groups were female samples only. Two studies did not state clearly how the sample of athletes was collected so it is hard to judge the quality of each study [35,42,45].

Summed up, the evidence seems to point towards no higher prevalence of pathogenic weight concerns or weight-control behaviour in athletes competing in leanness sports. However, this behaviour does seem to be particularly present in weight-class sports where success is often dependent on the class an athlete is competing in.

### Weight-control in connection with gender and age

Although it is suggested that female athletes are more susceptible to developing eating disorders or at higher risk for pathogenic weight-control behaviour than male athletes, one study by Artioli et al. did not find any gender differences [4]. They examined active judo competitors with the Rapid Weight Loss Questionnaire [46]. Their results showed that athletes lose up to 5% of their weight, mostly in connection with an upcoming competition. Increased exercises and restricted fluid intake were the favourite methods to lose weight. They did not find any differences between gender – neither in prevalence nor manifestation of pathogenic weight-control.

Looking at male athletes only, Galli and Reel [47] explored the body image and body enhancing behaviour of male athletes from various sports types in qualitative interviews. The findings show that male athletes want to reduce fat and gain muscle mass, desiring a more muscular, stronger body. An extreme version is the so-called “muscle dysmorphia”. Patients who suffer from it perceive their body as too slim or not muscular enough, even if there is no objective evidence [48]. It is a form of body dysmorphic disorder and it is referred to as “reverse anorexia” [49]. As a result of their perception, patients withdraw from social activities out of shame or stick to extensive workouts or usage of anabolic drugs [50]. In Galli’s study there was an interesting finding: Although 80% expressed some dissatisfaction regarding their physique and admitted to regularly use body-enhancing strategies (e.g. dieting, efforts to gain weight), 70% had some positive feelings about their bodies at the same time. This was mostly expressed as a confidence in their bodies’ abilities [47]. Clear strength of the study by Galli et al. is clearly the insight gained through qualitative data. However, this also leaves the potential risk of interviewer’s bias.

The majority of studies included in this review did find a higher frequency of pathogenic weight concerns and weight-control behaviour in female athletes than in male ones. Martinsen at al [13] could show in their study that female athletes have more concerns about their weight and a higher urge to improve their appearance than male athletes. Additionally, they used more pathogenic weight-control methods than their male counterparts. Johnson et al. [44] examined NCAA Division I athletes from 11 universities all over the States showing a higher frequency of some pathogenic weight-control behaviour in female athletes than in male ones independent of the type of sport. This was true for vomiting and use of laxatives as well as diet pills over a lifetime. However, no statistically significant difference could be shown for use of diuretics or steroids. Male athletes, on the other hand, were using the sauna or steam bath more often than females to lose weight, although this didn’t become significant. In general, female athletes wanted to reduce their body fat down to a percentage where they would stop menstruating risking osteoporosis within one year. Rouveix [35] at al examined 24 judo athletes and came to mixed conclusions. Nearly two thirds of the athletes lost more than 2.8 kg over the course of a season with no gender differences. Most common methods were intensification of exercising and reduction of fluid intake. There was a gender difference in methods to reduce weight, though: female athletes fasted significantly more. Interestingly, athletes reported their parents and society as equally important sources for the pressure to lose weight as their own drive to do so. Additionally, there was a clear gender difference for the ideal body with female athletes desiring a much lower body weight than male athletes. A definite strength of this study is its clear definition of the athlete level and a good control of the comparison group, who did not engage in sports activities more than three times a week.

Another interesting finding with regard to gender differences was found in the study of Rosendahl at al [34]: 50% of the examined female but just 10.1% of the male athletes wanted to reduce their weight and 19.6% of the male but just 3.4% of the female athletes wanted to gain weight. This confirms different mindsets and intentions behind weight-control behaviour in female and male athletes.

Only two studies also draw conclusions about the role of age in pathological weight concerns and weight-control behaviour. One study by Artioli et al. suggests that the earlier in life athletes started reducing weight before competition, the more aggressive they were with respect to their methods [4]. Another study from Marshall et al. [31] showed no significant difference in pathological weight concerns and weight-control behaviour between junior and senior field hockey players. However, both studies have their limitations: Whereas Artioli only asked athletes retrospectively where memories could be distorted; Marshall compared two different age groups in a cross-sectional study. Both did not examine athletes longitudinally to follow up the same cohort and their development.

In summary, a number of studies provide evidence for pathogenic weight-control behaviour in both genders. The form of pathogenic weight-control behaviour is different in male and female athletes. Whereas female athletes mostly want to reduce weight and focus on a slim appearance, male athletes mostly want to gain weight through muscle mass.

Concerning weight-control behaviour in connection with age, no uniform conclusion can be drawn, as only two studies looked at it at all. Results are heterogeneous, with one giving evidence that the age of an athlete starting with pathogenic weight-control behaviour might play a decisive role for the prognosis [4] whereas the other does not seem to see any connection with age [31].

### Discussion

To our best knowledge, this is the first systematic review on weight-concerns and weight-control behaviour in young elite athletes. It appears that young elite athletes are engaged in pathogenic weight-control behaviour despite long-term health being very important for the development of their sports career and achievement of peak performance.

Comparing athletes with non-athletes or controls, the majority of studies, several of which were large scale, found either no difference or an even lower risk of athletes for pathogenic weight concerns or weight-control behaviour [13,30,33-35,45]. Only one study could show a higher prevalence of pathogenic weight concerns but not weight-control behaviour in athletes [32].

Because of different demands associated with different sports types, we further analysed the studies found according to clusters. We decided to use the classification into leanness and non-leanness sports for the sub-analysis of different sports types, as this is a very common distinction first introduced by Sundgot-Borgen [51] in connection with eating disorders in elite athletes. Several of the studies included in our review used this particular classification. However, we are aware that there might be additional ways of clustering different types of sports that were not considered in this review. Focusing on leanness-sports, three studies give evidence of a higher prevalence of weight-control problems in leanness-sports [33,34,42]. However, four other studies did not find any differences in the prevalence between these two categories [13,18,19,32], and there was no study in which a higher prevalence of pathogenic weight-control behaviour in non-leanness sports could be shown. Whether leanness-sports are a risk factor for pathogenic weight-control or whether the higher prevalence is merely due to a selection bias has to be further investigated.

It was suggested that gender plays an important role for the prevalence of pathogenic weight-control behaviour. In total, only two studies made a clear statement about gender with contradictory results: whereas one study showed no difference between female and male athletes [4], the other one clearly supported the theory that female athletes are more engaged in pathogenic weight concerns and weight-control behaviour [13]. Two other studies showed mixed results within their different outcome variables [35,44]. Additional studies focus on female or male athletes alone, making gender comparison hard. However, they help to get a deeper understanding of gender specific ways to worry about body shape or weight-control.

Our review had to encounter several problems. In general, difficulties seem to arise when looking at the topic of pathogenic weight concerns and weight-control behaviour. Specifically, there does not seem to be a clear common definition of what weight concerns and weight-control behaviour are. As a result, a variety of different measuring instruments are used. This heterogeneity makes consistent conclusions difficult. In our review, we included all studies that used these two terms. However, further research should clarify what these terms comprise exactly to get a better idea of what appropriate measures might be. Another problem arises with choosing appropriate screening instruments. This is due to the fact that in some sports types the very same behaviours can be pathogenic or non-pathogenic depending on circumstances. Normally, screening instruments used are not specifically designed for or even adapted to athletes and their specific demands which might incorrectly classify non-pathogenic, functional behaviour as part of a pathogenic one [25]. Furthermore, the next difficult distinction is the thin line between pathogenic weight-control behaviour and eating disorders in elite sports. This is especially supported by the fact that in leanness-sports a very thin look is considered normal, and more radical forms of weight-control are often accepted by coaches and athletes [2,6,8,52]. Sundgot-Borgen even described a sport-specific variant of anorexia nervosa referred to as “anorexia athletica”. According to her, female athletes suffering from it show an intensive fear of gaining weight or even becoming obese, although the athlete is not overweight at all. To achieve their individually set ideal weight, these athletes use a variety of pathogenic weight-control techniques [8]. On the other hand, it is impossible to deny that for some sport disciplines a change of body weight is a necessary part of training and competition. Here, it is most crucial to support athletes in losing or gaining weight in a healthy way. Several organizations and associations have acknowledged this fact and now provide specific guidelines for healthy weight management in elite athletes [9,53]. However, particularly in elite sports, trainers’ experience and anecdotal knowledge plays an important role when it comes to the management of athletes’ concerns. Therefore it seems crucial to combine the two sources by providing trainers with additional information about the topic of weight concerns and weight-control behaviour in young elite athletes.

Another challenge for this review was the broad heterogeneity of studies. Their sample sizes ranged from 10 to 1445 participants. Some examined groups only consisted of athletes from a special type of sport, and some had no control-groups to give evidence of the differences between athletes and non-athletes Furthermore, results are mostly based on self-rating questionnaires and no expert interviews. This may have affected the validity, because many athletes might not answer truthfully or withhold their pathogenic concerns or weight-control behaviour. When some studies cooperated with the respective sports association or college, it cannot be excluded that athletes held back some information, as they might have been scared to lose privileges within their system. In any case, this would have only lead to an underestimation of the results found. Additionally, the methods differ strongly between cross-sectional studies with big cohorts and qualitative interview studies with small samples. Whereas, for example, Johnson et al. examined nearly 1500 athletes from 11 different universities [44], Galli and Reel examined 10 male athletes over ten in-depth semi-structured interviews regarding body image in male athletes [47]. Both methods are respectable forms of research but make a comparison difficult if not impossible. Finally, we can’t exclude publication bias.

## Conclusions

Taken together, the examined papers show that there is some prevalence of pathogenic weight concerns and weight-control behaviour in elite athletes. However, there is no certainty that the prevalence is higher than in control groups. Only in leanness-sports, where athletes are encouraged to be thin for either appearance or performance, the prevalence of pathogenic weight-control behaviour is higher in frequency than in non-athletes. The pressure of competitive sports seems therefore to be a risk factor for both genders.

Thus, special attention should be paid to athletes in leanness sports who have experiences with dieting and who show body- or weight-dissatisfaction.

Further research is necessary to get a better understanding of the connection between elite sports and pathogenic weight-control behaviour. This is why our research group examined pathogenic weight-control behaviour, body- and weight-dissatisfaction and subjective eating concepts in young elite athletes in the “GOAL-study” (“The German Young Olympic Athletes’ Lifestyle and Health Management Study“) [54] It consisted of a quantitative and a qualitative part and collected data from young squad athletes from all Olympic Disciplines allowing us to perform sub-analysis according to each single discipline. First results show that this combined approach provides comprehensive data [55-58]. In addition, long-term studies are needed to see what happens if young athletes stop competing as adults.

## Competing interests

The authors declare to have no competing interests.

## Authors’ contributions

AW and SZ carried out the literature search and drafted the manuscript. AT, JM and KEG have made substantial contributions to conception and design, and interpretation of data. All three have revised the article critically for important intellectual content. SS has made substantial contributions to interpretation of data. He has revised the article critically for important intellectual content. All authors have given final approval of the manuscript version to be published.
